# Ethylene Polymerization and Copolymerization with Polar Monomers Using Nickel Complexes Bearing Anilinobenzoic Acid Methyl Ester Ligand

**DOI:** 10.3390/polym10070754

**Published:** 2018-07-09

**Authors:** Hailong Cheng, Yue Su, Yanming Hu, Xuequan Zhang, Zhengguo Cai

**Affiliations:** 1 State Key Laboratory for Modification of Chemical Fibers and Polymer Materials, College of Material Science and Engineering, Donghua University, Shanghai 201620, China; chl_111@126.com (H.C.); 18817320298@163.com (Y.S.); 2 Key Laboratory of Synthetic Rubber, Changchun Institute of Applied Chemistry, Chinese Academy of Sciences, Changchun 130022, China; yanminghu@ciac.ac.cn (Y.H.); xqzhang@ciac.ac.cn (X.Z.)

**Keywords:** nickel catalyst, anilinobenzoic acid methyl ester, ethylene, polar monomer, copolymerization

## Abstract

Neutral nickel complexes containing an anilinobenzoic acid methyl ester ligand are prepared and applied for the ethylene polymerization and copolymerization with polar monomers. The complex **C2** containing isopropyl substituent on the aniline ligand conducts ethylene polymerization with high activity and good thermal stability. Most importantly, the catalyst promotes the copolymerization of ethylene and polar monomers with high activity (up to 277 kg·mol^−1^·h^−1^), affording ester-functionalized semicrystalline polyethylene with reasonable polar monomer content (up to 3.20 mol %).

## 1. Introduction

Although polyolefins have been extensively used in a wide range of application, the nonpolarity of polyolefins limits their further application [1]. This limitation could be dramatically improved by the introduction of even a small amount of polar functional groups to the polyolefin backbone [2]. A pioneering discovery was reported by Brookhart and co-workers that the α-diimine-nickel/palladium catalysts can initiate direct copolymerization of ethylene with polar monomers in the 1990s [3]. Since then, late-transition-metal catalysts based on palladium complexes have been extensively studied for olefin copolymerization with polar monomers [4,5,6,7,8,9,10,11]. Recently, low cost nickel catalysts were also developed extensively for the synthesis of functionalized polyolefins, although their copolymerization activities and resulting copolymer molecular weight are far from those required for industrial application [12,13,14,15,16,17,18,19,20].

**Chart 1:**
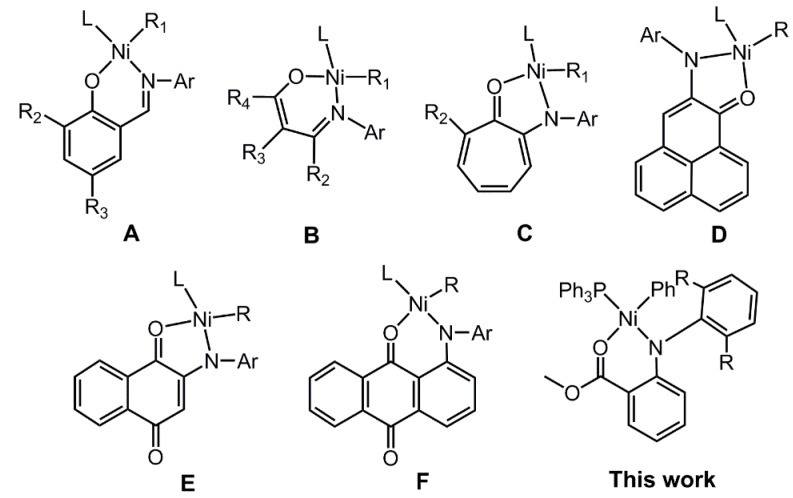
Representative [N,O] Ni Catalysts.

The significant interest was attracted in developing the neutral nickel catalysts because they exhibit higher tolerance toward functional polar groups [21]. Early examples of SHOP type neutral nickel catalysts have been commercialized for the synthesis of α-olefins [22,23,24]. A series of neutral nickel catalysts bearing salicylaldimine ligands (Chart 1**A**) was reported by Grubbs et al., which showed excellent tolerance towards polar monomers to give functionalized polyethylene [25,26,27,28]. The β-ketoiminato neutral nickel complexes with six-membered-ring were synthesized and applied as the olefin (*co*)polymerization catalyst (Chart 1**B**) [29,30,31,32,33,34,35,36,37]. Brookhart et al. successively developed the anilinotropone-based (Chart 1**C**) and anilinoperinaphtenone-based (Chart 1**D**) neutral nickel catalysts [38,39,40,41]. These nickel catalysts exhibited higher activity than salicylaldimine neutral nickel catalyst **A** in ethylene polymerization. Shiono et al. reported that five-membered anilinonaphthoquinone ligated neutral nickel complexes (Chart 1**E**) exhibited unique performances for the polymerization of ethylene and norbornene and copolymerization of ethylene with polar monomers [16,42,43,44,45]. Recently, we found that a novel six-membered anilinoanthraquinone-based neutral nickel complex promoted random copolymerization of ethylene with norbornene and polar monomers with high activity and good thermal stability (Chart 1**F**) [46,47]. Here, we report the synthesis, characterization, and application of six-membered neutral nickel complexes bearing anilinobenzoic acid methyl ester ligand for the ethylene polymerization and copolymerization with polar monomers to investigate the electronic effect of the ligand backbone.

## 2. Experimental Section

### 2.1. Materials

All experiments were performed under dry nitrogen atmosphere using standard Schlenk techniques or in a glovebox. All solvents were dried by the PS-MD-5 (Innovative Technology (China) Ltd., Hong Kong, China) solvent purification system. Ethylene was purified by passage through dehydration column of ZHD-20 and deoxidation column of ZHD-20A before using. Commercial polar monomers were distilled ov`er calcium hydride before using. The other reagents were purchased and used without purification.

### 2.2. Analytical Procedure

NMR spectra were measured on a Bruker Asend^TM^ 600 spectrometer (Bruker, Karlsruhe, Germany). CDCl_3_ was employed as solvent and the central peak of the solvent was used as an internal reference (CDCl_3_, 7.26, {77.16} ppm). Differential scanning calorimeter (DSC) analyses were carried out on a TA Q2000 instrument (Waters, New Castle, DE, USA). The DSC curves of the samples were recorded under a nitrogen atmosphere at a heating rate of 10 °C·min^−1^. The single crystals data was made on a Bruker APEX2 diffractometer (Bruker, Karlsruhe, Germany) using graphite monochromated with Mo Kα radiation (*l* = 0.71073 Å), and the measurement were mounted under nitrogen atmosphere at low temperature. Crystallographic data are summarized in Table S1. Molecular weight and molecular weight distribution of polymers obtained were tested by a polymer laboratory PL GPC-22 (Agilen, Santa Clara, CA, USA) at 150 °C using 1,2,4-trichlorobenzene as a solvent.

### 2.3. General Polymerization Procedure

Atmospheric pressure polymerization was carried out in a 100 mL glass reactor equipped with a magnetic stirrer and the high pressure polymerization was performed in Parr Instrument Company autoclave. At first, the reactor was vacuumized at 110 °C and charged with nitrogen several times before polymerization procedure. Then the prescribed amounts of toluene, monomers and cocatalyst under nitrogen were charged into the reactor and polymerization was initiated by adding the catalyst solution at desired temperature and desired pressure. The polymerization was conducted for a certain time and terminated with acidic methanol. The polymer obtained was stirred overnight, collected by filtration, adequately washed with alcohol and acetone, and dried under vacuum at 80 °C overnight until a constant weight was reached.

### 2.4. Synthesis of Ligands and Complexes

#### 2.4.1. Synthesis of 2-Anilinobenzoic Acid Methyl Ester Ligand **L1**

A solution of bis(dibenzylideneacetone) palladium (Pd(dba)_2_) (0.230 g, 0.4 mmol), 1,1′-bis(diphenylphosphino)ferrocene (dppf) (0.333 g, 0.6 mmol), Methyl 2-bromobenzoate (1.42 mL, 10 mmol), aniline (1.1 mL, 12 mmol) and CsCO_3_ (4.89 g, 15 mmol) in toluene (100 mL) was heated to 110 °C and stirred for 24 h to afford a brown solution. The solvent was evaporated under reduced pressure, and then the residue was dissolved in dichloromethane. The organic phase was washed with water (20 mL × 5) and dried over anhydrous sodium sulfate. Then the solvents were filtrated and evaporated under reduced pressure. The residue l was extracted with ether and washed three times by 20 mL hexane to yield brown oil. Yield 2.091 g (92%). ^1^H NMR (CDCl_3_) (Figure S1): δ = 9.47 (s, 1H, –N*H*), 7.98–7.96 (d, *J* = 9.3 Hz, 1H, aryl–*H*), 7.36–7.30 (m, 3H, aryl–*H*), 7.26–7.24 (m, 3H, aryl–*H*), 7.11–7.08 (t, *J* = 7.3 Hz, 1H, aryl–*H*), 6.75–6.72 (t, *J* = 7.4, 7.52 Hz, 1H, aryl–*H*), 3.91 (s, 3H, –OC*H*_3_). ^13^C NMR (CDCl_3_) (Figure S2): δ 168.90, 147.93, 140.77, 134.10, 131.54, 129.38, 123.54, 122.46, 117.11, 114.00, 111.89, 51.74. Elemental Analysis: C_14_H_13_NO_2_: Calcd. C 73.99, H 5.77, N 6.16; Found: C 73.34, H 5.35, N 6.23. Elemental Analysis: C_14_H_13_NO_2_: Calcd. C 73.99, H 5.77, N 6.16; Found: C 73.34, H 5.35, N 6.23.

#### 2.4.2. Synthesis of 2-(2,6-Diisopropylaniline)-Methyl Benzoate Ligand **L2**

Ligand **L2** was synthesized in a similar way to that for ligand **L1**. **L2** was recrystallized in hexane to yield a reddish crystal. Yield 2.62 g (84%). ^1^H NMR (CDCl_3_) (Figure S3): δ 9.03 (s, 1H, –N*H*), 7.98-7.97 (dd, *J* = 8.0, 1.5 Hz, 1H, aryl–*H*), 7.38–7.30 (t, 1H, aryl–*H*), 7.28–7.23 (m, 2H, aryl–*H*), 7.20–7.17 (m, 1H, aryl–*H*), 6.63–6.60 (td, *J* = 7.6, 0.9 Hz, 1H, aryl–*H*), 6.22–6.21 (d, *J* = 8.5 Hz, 1H, aryl–*H*), 3.95 (s, 3H, –OC*H*_3_), 3.17–3,10 (hept, *J* = 6.8 Hz, 2H, –C*H*), 1.19–1.12 (dd, *J* = 34.4, 6.9 Hz, 12H, –C*H*_3_). ^13^C NMR (CDCl_3_) (Figure S4): 185.61, 183.97, 155.70, 150.10, 135.23, 134.40, 134.33, 134.02, 133.30, 133.13, 127.07, 127.03, 126.84, 121.49, 117.27, 116.53, 114.14, 104.58, 56.08. Elemental Analysis: C_20_H_25_NO_2_: Calcd. C 77.14, H 8.09, N 4.50; Found: C 77.47, H 7.92, N 4.21. ^13^C NMR (CDCl3) δ 169.41, 151.51, 147.80, 134.48, 134.45, 131.49, 127.80, 124.01, 115.39, 113.03, 109.59, 77.37, 77.16, 76.95, 51.72, 28.52, 24.74, 23.14, 0.13.

#### 2.4.3. Synthesis of Nickel Complex **C1**

A mixture of the ligand **L1** (0.227 g, 1 mmol) and KH (0.044 g, 1.1 mmol) in 50 mL of THF was stirred for 3 h to afford potassium salts of **L1**. The THF solution of potassium salts was slowly dropped into a solution of trans-[Ni(PPh_3_)_2_ PhCl] (0.696 g, 1 mmol) in 5 mL of the same solvent. The mixture was stirred over night at 25 °C. The reaction mixture was filtered off under a nitrogen atmosphere (PTFE, 0.45 μm) and evaporated under vacuum. The solid thus obtained was purified by recrystallization with a mixture of toluene/hexane to afford crimson single crystals. Yield 0.305 g (49%). Elemental Analysis: C_38_H_32_NNiO_2_P, Calcd. C 73.10, H 5.17, N 2.24; Found: C 73.48, H 5.20, N 2.34.

#### 2.4.4. Synthesis of Nickel Complex **C2**

Complex **C2** was synthesized in a similar way to that for Complex **C1**. **C2** was purified by recrystallization with a mixture of THF/hexane to afford orange-red single crystals. Yield 0.291 g (41%). Elemental Analysis: C_44_H_44_NNiO_2_P, Calcd. C 74.59, H 6.26, N 1.98; Found:C 74.37, H 6.29, N 2.08.

## 3. Results and Discussion

### 3.1. Synthesis and Molecular Structure of Nickel Complexes

**Scheme 1:**
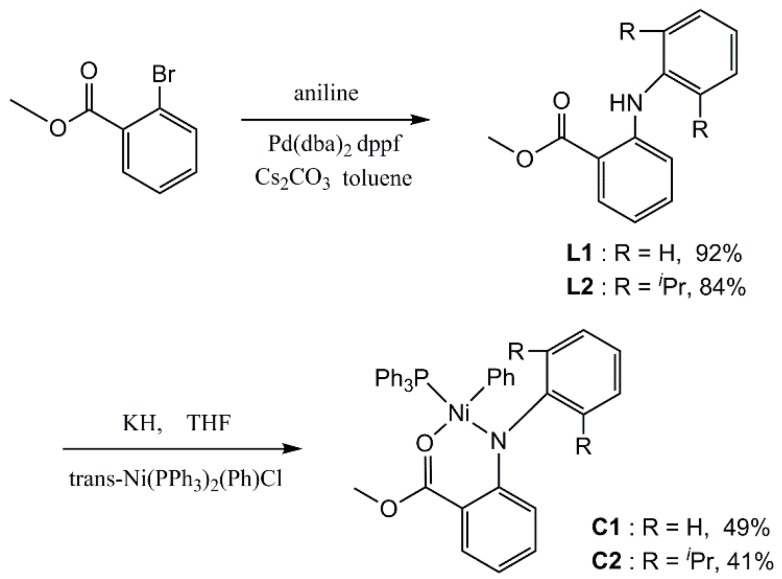
Synthesis of the Anilinoanthracenequinone Ligands and the Corresponding Nickel Complexes.

The synthesis procedure of the ligands **L1**–**L2** and the corresponding nickel complexes **C1**–**C2** is shown in Scheme 1. 2-Anilinobenzoic acid methyl ester ligands were easily obtained by the amination of methyl 2-bromobenzoate in the presence Pd(dba)_2_/dppf and Cs_2_CO_3_ [48]. The nickel complexes were prepared from the reaction of corresponding potassium salt of ligand with 1 equiv of *trans*-[Ni(PPh_3_)_2_PhCl] in good yields according to the literature procedure [49].

**Figure 1:**
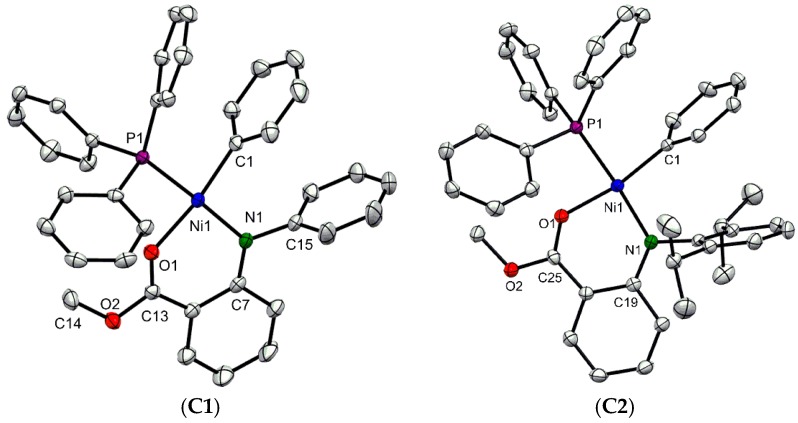
Molecular structures of complexes **C1** and **C2**. H atoms are omitted for clarity. Selected bond lengths (Å) and angles (deg) for **C1**: Ni1-N1 = 1.9223 (14), Ni-C1 = 1.8841 (17), Ni1-O1 = 1.9180 (12), Ni1-P1 = 2.1758 (5), O1-Ni1-N1 = 91.14 (6), C1-Ni1-P1 = 87.12 (5); **C2**: Ni1-N1 = 1.932 (2), Ni-C1 = 1.891 (2), Ni1-O1 = 1.9193 (17), Ni1-P1 = 2.1816 (7), O1-Ni1-N1 = 91.33 (8), C1-Ni1-P1 = 86.59 (7).

The molecular structures of nickel complexes were determined by single-crystal X-ray diffraction analysis (Figure 1). Complexes **C1** and **C2** showed very similar structure in view of the bond distances and the bond angles and exhibited a six-membered (N, O) nickel chelate ring with a puckered conformation. Each nickel center adopted a square planar coordination geometry, and the phosphine occupied the trans position to the nitrogen.

### 3.2. Ethylene Polymerization

The nickel complexes **C1** and **C2** were applied in ethylene polymerization. The results are summarized in Table 1. **C1** containing nonsubstituted aniline ligand was inactive regardless of the cocatalyst used. In contrast, **C2** containing isopropyl substituent on the aniline ligand in the presence of trialkylaluminum-free dried modified methylaluminoxane (dMMAO) or Ni(COD)_2_ promoted ethylene polymerizaiton with almost the same activities. The results suggested that the substituent on the aniline ligand and the cocatalyst as a phosphine scavenger are necessary for ethylene homopolymerization. The similar effects of the ligand substituent were observed with both cationic and neutral nickel catalyzed ethylene polymerization [3,4,5,6,7,8,9,10]. The Ni(COD)_2_ system gave high molecular-weight polyethylene (up to 76.3 kg·mol^−1^) with narrow molecular weight distribution, while the dMMAO system produced ethylene oligomers along with a small amount of polyethylene (entry 2, Table 1). The improved chain transfer rate in the dMMAO system may be attributed to the efficient dissociation of the phosphine, owing to the stronger Lewis acidity of dMMAO than that of Ni(COD)_2_. The same phenomenon was also reported by Kim et al. by using ketoenamine-based neutral nickel complexes, in which the use of MAO conducted ethylene oligomerization with high activity [50].

**Figure 2:**
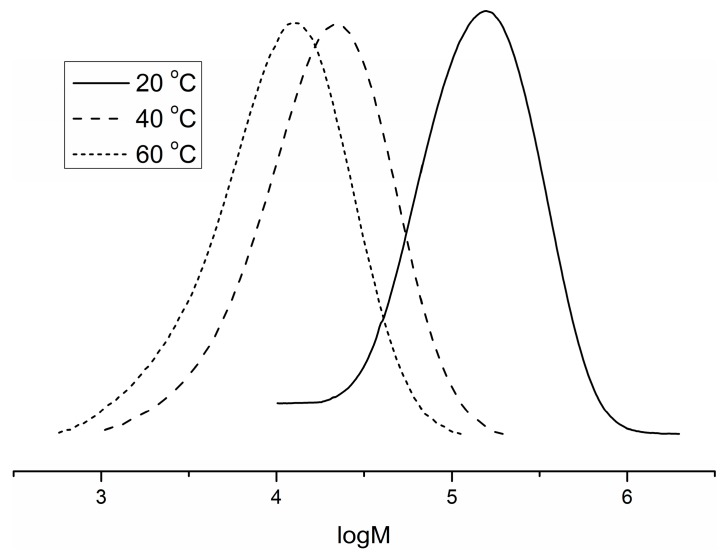
GPC curves of polymers under different polymerization temperatures.

Polymerization activity is strongly depended on the polymerization temperature and the ethylene pressure. The decrease of the temperature to 20 °C resulted in the decrease of the activity in one order of magnitude (entry 5). Although activity decreased as the temperature was increased to 60 °C (entry 6), the catalyst still showed high activity of 336 kg·mol^−1^·h^−1^, indicating good thermal stability of this catalytic system. The reason for the small decrease of the activity at 60 °C could be attribute to the reduced ethylene concentration at the high polymerization temperature [51]. The molecular weight increased with decreasing the polymerization temperature, indicating that the increase of chain transfer rate is more effective than chain growth rate with rising the polymerization temperature (Figure 2). Further increase in activity and molecular weight was observed by the increase of ethylene pressure at 40 °C.

### 3.3. Copolymerization of Ethylene with Polar Monomers

The ethylene copolymerizations with polar monomers such as 5-hexene-1-yl-acetate (HAc), 5-norbornene-2-yl acetate (NB_Ac_), vinyl acetate (VAc), and methyl acrylate (MA) were conducted (Table 2). Although the Ni(COD)_2_ system showed slightly higher comonomer incorporation than the dMMAO system, the copolymerization activity of Ni(COD)_2_ system was much lower than that of the dMMAO system, affording lower molecular weight copolymers (entries 1 and 3). In general, the polymerization of the sterically bulky monomers such as higher α-olefins and norbornene catalyzed by the neutral nickel complexes require MAO or MMAO as the phosphine scavenger to provide reasonable monomer coordination site [10].

**Figure 3:**
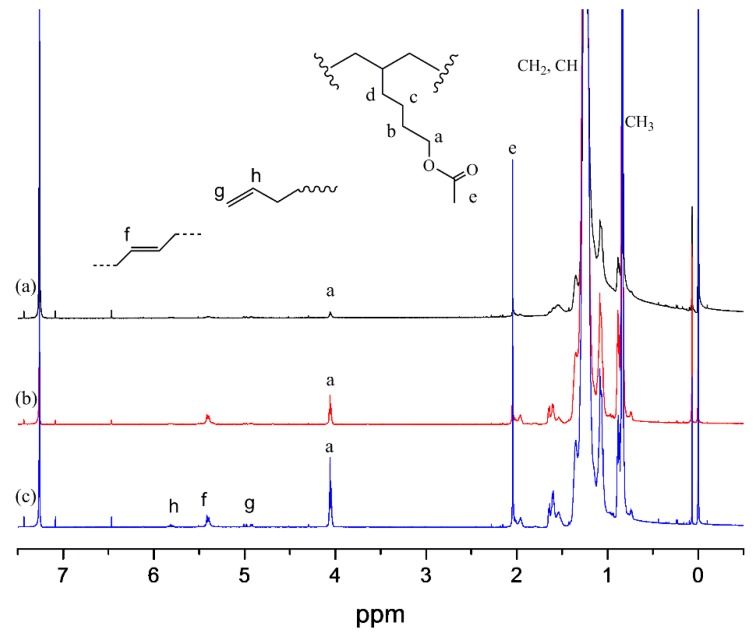
^1^H NMR spectra of the E-HAc copolymers: (**a**) 0.35 mol %; (**b**) 0.88 mol %; (**c**) 1.33 mol %.

The incorporation of the copolymers obtained was investigated by ^1^H NMR, and the typical ^1^H NMR spectra of copolymers obtained by complex **C2** are illustrated in Figure 3. The signals at 4.06 ppm and 2.05 ppm in copolymers were assigned for C*H*_2_ and C*H*_3_ protons of ester-group label as a and e, respectively. The microstructure of the E-HAc copolymer was also investigated by ^13^C NMR to demonstrate the incorporation of polar monomers (Figure S5) [16,52]. HAc incorporation was increased (up to 1.33 mol %, entry 4) by increasing HAc concentration in the feed, although copolymerization activity and copolymer molecular weight were decreased slightly. As compare to the results with complex **F** [47], anilinobenzoic acid methyl ester-ligated complex **C2** showed slightly lower copolymerization activity and HAc incorporation.

The nickel complex **C2** was also capable of copolymerizing ethylene with 5-norbornene-2-yl acetate (NB_Ac_) with good activity, generating E-NB_AC_ copolymer with reasonable incorporation (3.20 mol %, in Figure S6)). Unfortunately, the commercial polar comonomers such as VAc, and MA shut down copolymerization activity (entries 6, 7). This was mainly caused by the coordination of the carbonyl oxygen atom to the nickel center to generate a stable six-membered nickel chelate compound [4].

## 4. Conclusions

In conclusion, neutral nickel complexes bearing anilinobenzoic acid methyl ester ligand have been synthesized and characterized. The steric effect on the aniline ligand played a significant role in the polymerization performances, in which isopropyl substituted complex **C2** could enhance ethylene polymerization activity. The complex **C2** was also able to promote copolymerization of ethylene with polar monomers with high activity (114–277 kg·mol^−1^·h^−1^) and reasonable polar monomer incorporation.

## Figures and Tables

**Table 1:** Ethylene polymerizations with complexes **C1** and **C2** ^a^.

Entry	Catalyst	Cocatalyst	P (MPa)	Temp (°C)	Yield (g)	Activity (kg·mol^−1^·h^−1^)	*M*_n_^b^ (10^3^)	PDI ^b^	*T*_m_^c^ (°C)
1	**C2**	-	1.5	40	0.06	36	10.4	2.14	97.0
2 ^d^	**C2**	dMMAO	1.5	40	oligomer	616	-	-	-
3	**C1**	Ni(COD)_2_	1.5	40	trace	-	-	-	-
4	**C2**	Ni(COD)_2_	1.5	40	1.29	774	12.2	2.08	90. 6
5	**C2**	Ni(COD)_2_	1.5	20	0.14	84	76.3	2.28	125.9
6	**C2**	Ni(COD)_2_	1.5	60	0.56	336	6.9	2.11	45.2
7	**C2**	Ni(COD)_2_	0.5	40	0.17	99	8.6	1.85	87.3
8	**C2**	Ni(COD)_2_	3.0	40	1.46	876	19.1	2.54	95.2

^a^ Polymerization conditions: solvent = toluene, total volume = 25 mL, Ni = 10 μmol, time = 10 min, temperature = 40 °C, Ni(COD)_2_ = 24 μmol, dMMAO = 10 mmol. ^b^ Determined by GPC using polystyrene standard. ^c^ Determined by DSC. ^d^ Activity was determined by ^1^H NMR.

**Table 2:** Copolymerization of ethylene with polar monomers using complex **C2** ^a^.

Entry	Comonomer (mmol)	Cocatalyst	Yield (g)	Activity (kg·mol^−1^·h^−1^)	*M*_n_^b^ (10^4^)	PDI ^b^	*T*_m_^c^ (°C)	Incorp. ^d^ (mol %)
1	HAc(30)	Ni(COD)_2_	0.05	5	0.87	1.92	97.8	1.01
2	HAc(10)	dMMAO	2.77	277	1.97	2.00	97.9	0.35
3	HAc(30)	dMMAO	2.01	201	1.31	2.39	78.1	0.88
4	HAc(60)	dMMAO	1.74	174	1.17	2.16	71.4	1.33
5	NB_Ac_ (30)	dMMAO	1.14	114	1.52	3.21	67.1	3.20
6	VAc(30)	dMMAO	trace	-	-	-	-	-
7	MA(30)	dMMAO	trace	-	-	-	-	-

^a^ Polymerization conditions: solvent = toluene, total volume = 25 mL, Ni = 10 μmol, pressure = 15 atm, temperature = 40 °C, time = 1 h, dMMAO = 10 mmol, Ni(COD)_2_ = 24 μmol. ^b^ Determined by GPC using polystyrene standard. ^c^ determined by DSC. ^d^ Incorporation determined by ^1^H NMR.

## Supplementary Materials

The following are available online at http://www.mdpi.com/2073-4360/10/7/754/s1, Figure S1: ^1^H NMR spectrum of ligand **L1**, Figure S2: ^13^C NMR spectrum of ligand **L1**. Figure S3: ^1^H NMR spectrum of ligand **L2**, Figure S4: ^13^C NMR spectrum of ligand **L2**. **Figure S5**. ^13^C NMR spectrum (CDCl_3_, 50 °C) of E-HAc copolymer by **C2**-dMMAO (entry 4, Table 2). Figure S6: ^1^H NMR spectrum (CDCl_3_) of E-NB_-Ac_ copolymer by C**2**-dMMAO (entry 5, Table 2). Table S1: Crystal data and structure refinement for complex **C1** and complex **C2**.
