# Supplementary material for: Ethylene Polymerization and Copolymerization with Polar Monomers Using Nickel Complexes Bearing Anilinobenzoic Acid Methyl Ester Ligand
Source: Polymers (Basel). 2018 Jul 9;10(7):754. doi: 10.3390/polym10070754 (PMC6403595; doi:10.3390/polym10070754)
Supplement: Supplementary file 1 [file polymers-10-00754-s001.zip › polymers-317165.supplementary.proofdone/Supplementary Materials.docx]

**Supplementary Materials**

**Ethylene Polymerization and Copolymerization with Polar Monomers Using Nickel Complexes Bearing Anilinobenzoic Acid Methyl Ester Ligand**

**Hailong Cheng^1^, Yue Su^1^, Yanming Hu^2^, Xuequan Zhang^2^, Zhengguo Cai^1,^***

^1^ State Key Laboratory for Modification of Chemical Fibers and Polymer Materials, College of Material Science and Engineering, Donghua University, Shanghai 201620, China; chl_111@126.com (H.C.); 18817320298@163.com (Y.S.)

^2^ Key Laboratory of Synthetic Rubber, Changchun Institute of Applied Chemistry, Chinese Academy of Sciences，Changchun 130022, P. R. China; yanminghu@ciac.ac.cn (Y.H); [xqzhang@ciac.ac.cn](mailto:xqzhang@ciac.ac.cn) (X.Z.)

Corresponding Author

*E-mail: [caizg@dhu.edu.cn](mailto:caizg@dhu.edu.cn)

1. **Chracterization of Ligands and Catalysts**


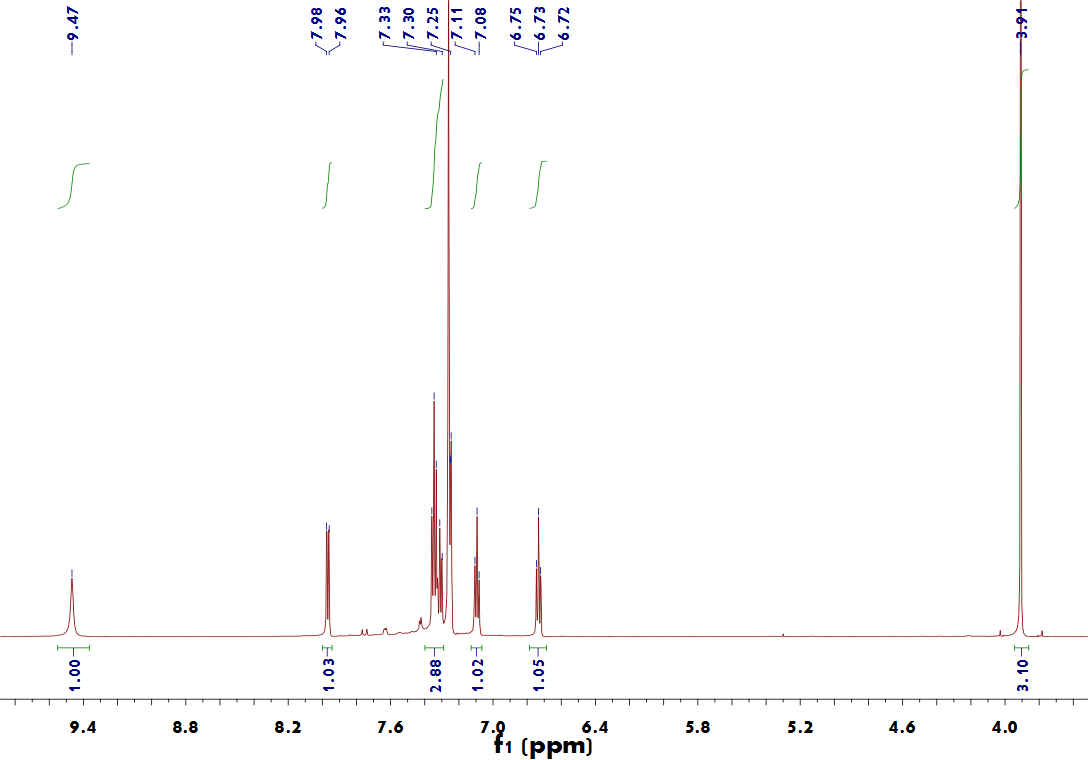


**Figure S1**. ^1^H NMR spectrum of L1.


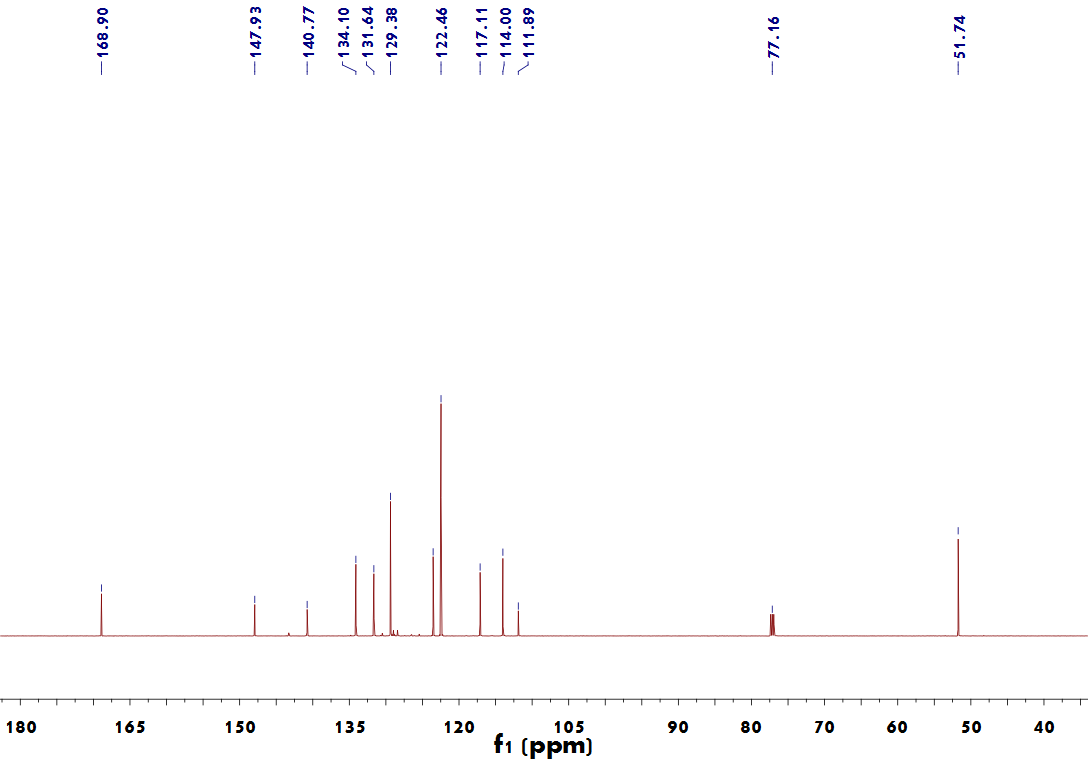


**Figure S2**. ^13^C NMR spectrum of L1.


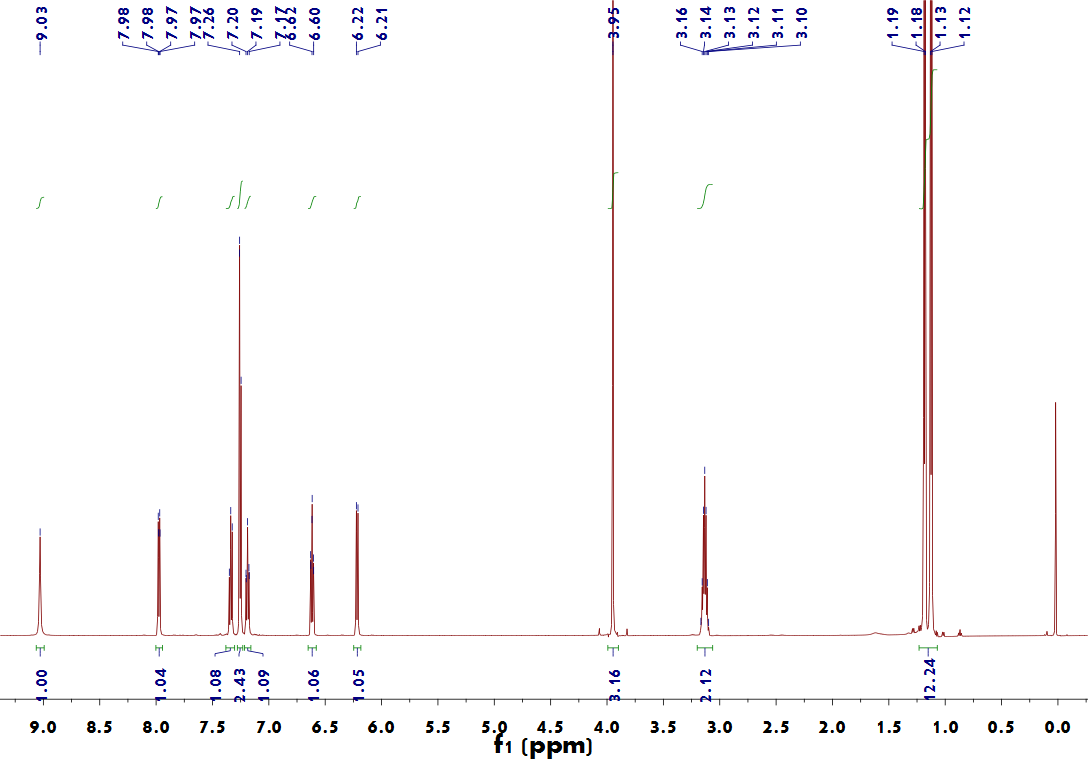


**Figure S3**. ^1^H NMR spectrum of L2.


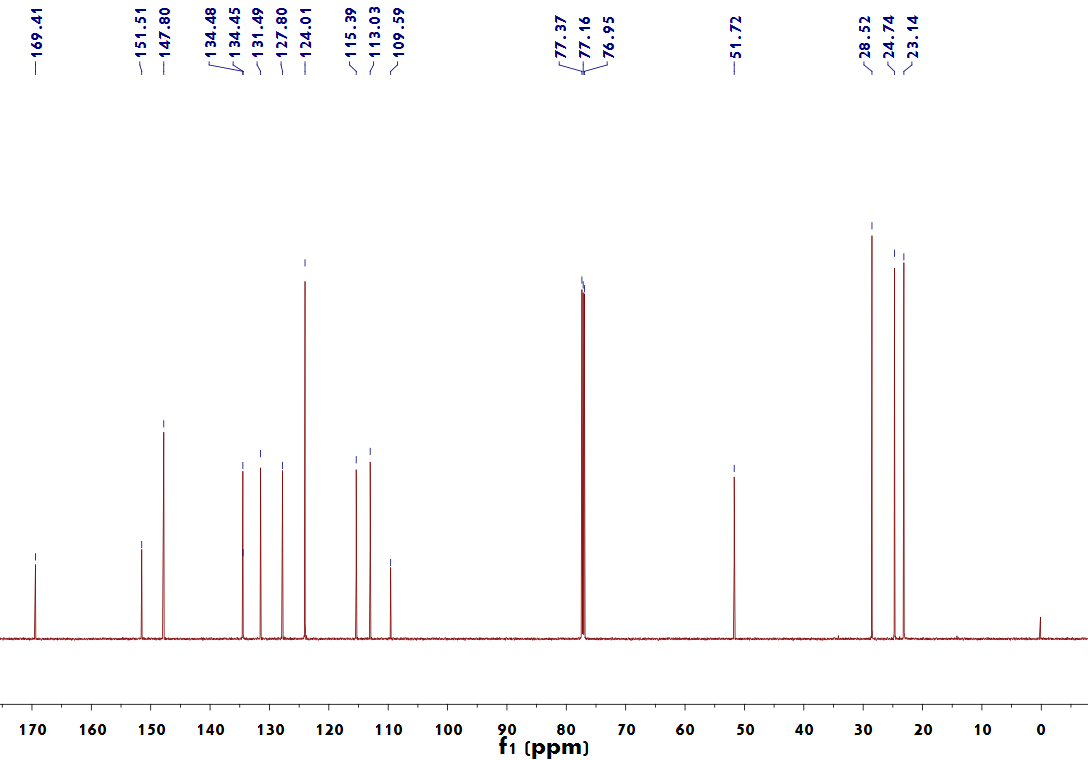


**Figure S4**. ^13^C NMR spectrum of L2.

**Table S1.** Crystal data and structure refinement for complex **C1** and complex **C2**.

| Complex | **C1** | **C2** |
| --- | --- | --- |
| Empirical formula  formula weight  Temperature  Wavelength  crystal system  space group  Unit cell dimensions  a (Å)  b (Å)  c (Å)  Volume  Z  Density (calculated)  Absorption coefficient  F(000)  Crystal size  Theta range for data collection  Index ranges  Reflections collected  Independent reflections  Completeness to theta = 26.000°  Absorption correction  Max. and min. transmission  Refinement method  Data / restraints / parameters  Goodness-of-fit on F^2^  Final R indices [I>2sigma(I)]  R indices (all data)  Extinction coefficient  Largest diff. peak and hole | C_38_ H_32_ N Ni O_2_ P  624.32  130 K  0.71073 Å  Monoclinic  P 1 21/n 1  a = 11.1866(8) Å  b = 19.7986(14) Å  c = 13.9483(11) Å  3084.1(8) Å3  4  1.345 Mg/m3  0.716 mm-1  1304  0.25 x 0.2 x 0.15 mm3  1.788 to 30.794°.  -16<=h<=16, -28<=k<=26, -20<=l<=18  31337  9568 [R(int) = 0.0512]  100 %  Semi-empirical from equivalents  0.7461 and 0.6852  Full-matrix least-squares on F^2^  9298 / 0 / 500  1.009  R1 = 0.0384, wR2 = 0.0779  R1 = 0.0753, wR2 = 0.0898  n/a  0.344 and -0.547 e.Å-3 | C_44_ H_44_ N Ni O_2_ P  708.48  273.15 K  0.71073 Å  Triclinic  P -1  a = 11.9217(10) Å  b = 16.4412(13) Å  c = 19.0949(16) Å  3617.6(5) Å3  4  1.301 Mg/m3  0.619 mm^-1^  1496  0.2 x 0.1 x 0.08 mm3  1.262 to 30.785°.  -17<=h<=15, -23<=k<=23, -27<=l<=25  37286  22193 [R(int) = 0.0487]  99.9 %  Semi-empirical from equivalents  0.7461 and 0.6810  Full-matrix least-squares on F^2^  22193 / 0 / 893  0.998  R1 = 0.0519, wR2 = 0.1026  R1 = 0.1165, wR2 = 0.1258  n/a  0.572 and -0.774 e.Å-3 |

**2. Characterization of Polymers**


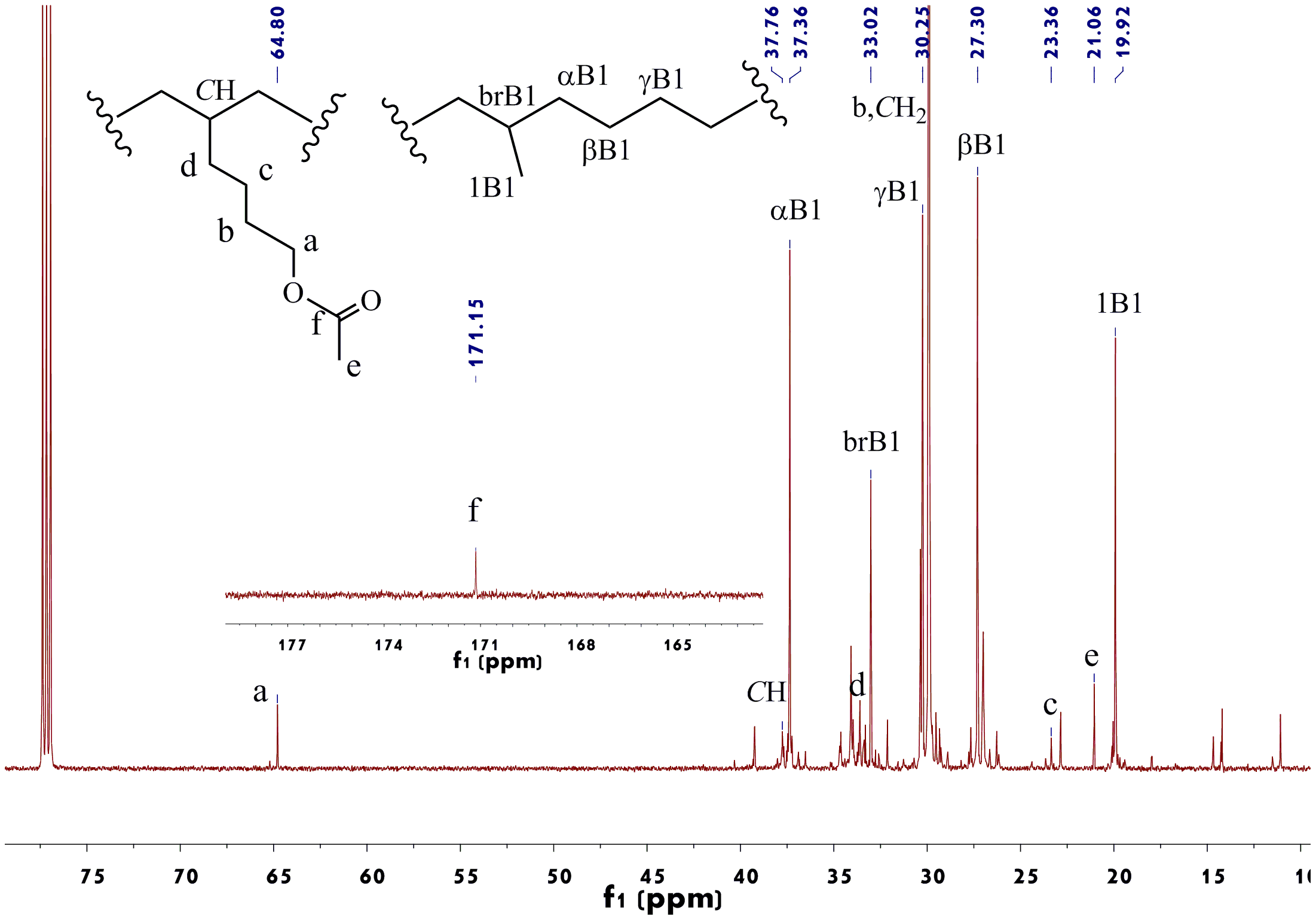


**Figure S5**. ^13^C NMR spectrum (CDCl_3_, 50 °C) of E-HAc copolymer by C**2**-dMMAO (entry 4, Table 2).


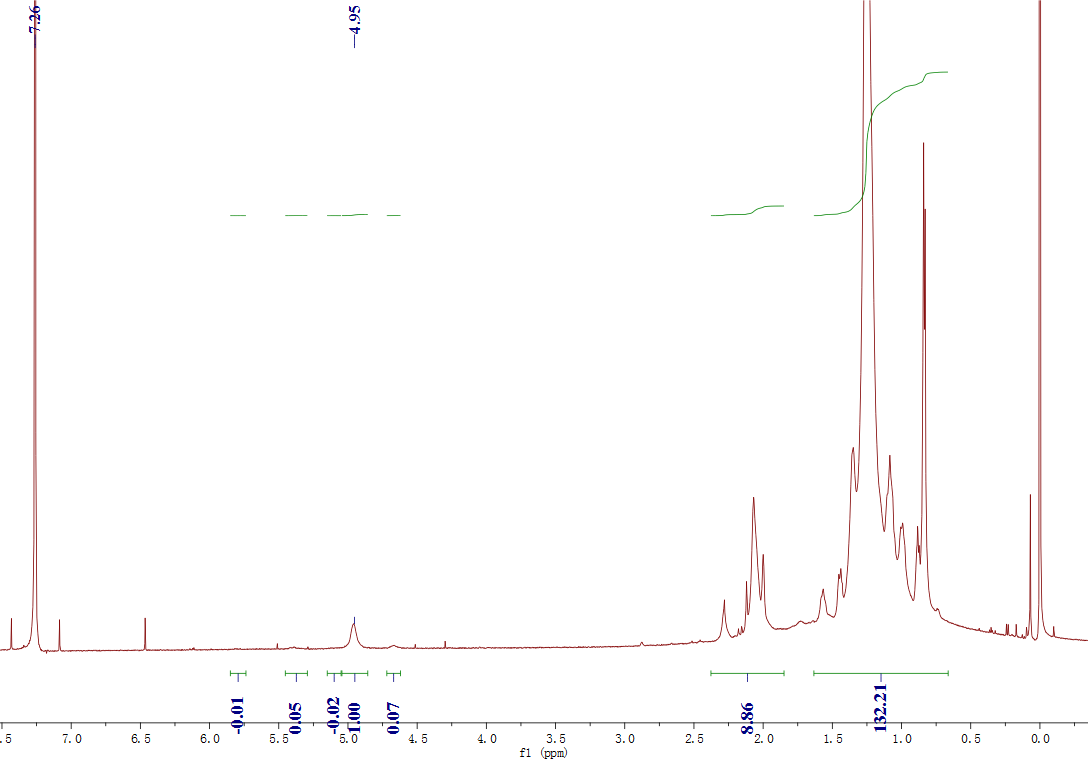


**Figure S6**. ^1^H NMR spectrum (CDCl_3_) of E-NB_-Ac_ copolymer by C**2**-dMMAO (entry 5, Table 2).
